# Vaccine Hesitancy and Acceptance: The Present and the Future

**DOI:** 10.3390/vaccines13010031

**Published:** 2024-12-31

**Authors:** Sherri Sheinfeld Gorin

**Affiliations:** 1 Department of Family Medicine, School of Medicine, University of Michigan, Ann Arbor, MI 48104, USA; ssgorin@med.umich.edu or sherri.gorin@gmail.com; 2 Joint Appointment, School of Public Health, University of Michigan, Ann Arbor, MI 48104, USA

In the following Special Issue of *Vaccines*, entitled “Acceptance and Hesitancy in Vaccine Uptake,” seventeen diverse papers examine the critical role of vaccination in health promotion and disease prevention. Immunization is key to primary healthcare and a cost-effective investment. Vaccines are also critical to the prevention and control of infectious disease outbreaks, as evidenced by the COVID-19 pandemic. The pandemic strained health systems and undermined health services, resulting in increased worldwide morbidity and mortality [1]. In 2019, the World Health Organization listed vaccine hesitancy as one of the ten threats to global health [2].

The papers included in this Special Issue explore the various influences on vaccine hesitancy, booster hesitancy, intention to vaccinate, and uptake against numerous preventable illnesses in low-risk children, adolescents, young adults, and those at higher medical risk. The vaccines described in these papers are not comprehensive and focus primarily on COVID-19 vaccination hesitancy or pre- or post-pandemic influences on other vaccinations. Yet, these papers offer general findings to mitigate the challenges of vaccine hesitancy worldwide.

Across all of the studies included in this Special Issue [3,4,5,6,7,8,9,10,11,12,13,14,15,16,17,18,19,20], the most frequent vaccinations were those for influenza (N = 7 papers), COVID-19 (N = 5 papers), adolescent vaccines N = 2), mpox, polio, and routine vaccinations for those age two and under (N = 1 each). Vaccinations for those age two and under included those against bacillus Calmette- Guérin, poliovirus, pneumococcus, diphtheria, tetanus, pertussis, hepatitis B, Hemophilus influenzae type B, measles–rubella combination, and rotavirus. The study findings were drawn from across the globe, with those from China (N = 4, including Hong Kong) most frequent, followed by those from the USA (N = 3) and Jordan (N = 2); those from Yemen, Greece, Malaysia, Pakistan, Africa, Spain, Canada, and other countries around the globe (N = 1 each) were less frequent. Studies included children, adolescents, young adults including college students, parents of children (including those diagnosed with diabetes), and high-risk adults (e.g., those diagnosed with diabetes, men who have sex with men, and healthcare workers who work on hospital wards with high rates of seasonal influenza), refugees, and older adults. The methods and data sources used varied as well, with cross-sectional surveys most frequent (N = 10), generally at the population level. Five of these surveys were administered online. Four cross-sectional surveys were administered to specific populations, such as those in residential care homes. Secondary data analysis, including from the US National Immunization Survey—Teens [NIS-T], founded three studies. Two of these papers relied on machine learning approaches as well. One paper rested on a randomized clinical trial, and one relied on qualitative interviews; one systematic review used a narrative synthesis approach, and one, a meta-analysis.

Only one paper reported the findings from an interventional study with messages about either the safety or effectiveness of COVID-19 mRNA boosters versus a control group. The authors of only one paper based their study on a theoretical model—the Health Belief Model [21].

Two of these papers were systematic reviews. The authors of one systematic review of 13 quantitative studies summarized the willingness of adolescents aged 10 to 19 years of age to receive the COVID-19 vaccine and the factors influencing their decision. An additional systematic review of 41 studies from fourteen African countries examined communication strategies for 13 different vaccines, primarily those administered to children.

In general, the findings emphasized the influence of sociodemographic factors (such as age and gender), perceived benefits and barriers, systematic data collection and analysis across populations and high-risk subgroups, and the importance of interventions to address vaccine hesitancy and improve uptake. Six general themes describe the influences on vaccination across countries and vaccination types across these 17 papers:

*Sociodemographic factors:* Older teens, college-aged students, and the elderly were more vaccine hesitant than others; lower vaccination rates are observed among non-Hispanic Black individuals compared to White individuals in the US. Unemployment was related to higher vaccine hesitancy, as was the level of education. Mothers were less hesitant than fathers for their children to undergo influenza vaccination. Spanish college students in non-health-related fields held less favorable attitudes and beliefs toward vaccination than other similar students.

*Perceived risks and benefits of vaccination:* A better understanding of the benefits of vaccination tended to reduce vaccine hesitancy. Privacy concerns were notable barriers, particularly for mpox vaccination. Among athletes, time constraints were a major barrier to vaccination. Parents of young children who expressed misconceptions about vaccine safety and efficacy raised significant barriers to flu vaccine acceptance.

*Motivational factors* increased vaccine hesitancy: These factors included cues to action such as the awareness of severe cases and perceived risk due to existing chronic conditions. Among healthcare workers, vaccine acceptance was linked to perceived susceptibility and benefits. These factors align well with the predictions of the Health Belief Model [21].

*(5) Cultural and medical risk population subgroups:* Among refugees, hesitancy was influenced by trust in intermediaries and culturally responsive healthcare. Diabetic patients showed higher hesitancy related to the duration of their condition. Political conservatives experienced greater vaccine hesitancy than others. With the second COVID-19 booster, older adult vaccine hesitancy was influenced by adverse events and deaths among close contacts.

*(6) Public Health Emergencies* negatively affected vaccination rates in regions such as Coastal Hadhramaut (Yemen) and within refugee populations. Within areas experiencing conflict across Pakistan, integrating community engagement with maternal and child health services through health camps significantly enhanced immunization coverage.

*(6) Educational and Community-Based Interventions* were key to addressing misconceptions about vaccination safety and its benefits. Healthcare communication that provided clear, consistent information about the flu vaccine’s safety and benefits was vital to help enhance vaccine uptake. Educational approaches that included scientific explanations increased the acceptance of vaccination among those with varied political views. Among Chinese MSM, higher mpox-related knowledge reduced vaccine hesitancy. Positive messaging increased the willingness to vaccinate and reduced vaccine hesitancy when messengers were transparent and trustworthy.

Similarly, trust-building was found to be key to implementing vaccine communications in a systematic narrative review of vaccine interventions across Africa. Community engagement was an essential part of these approaches to build trust. While mass vaccination campaigns in Africa were most common, some educational interventions targeted health workers to improve their interpersonal communication skills with patients. Both direct (e.g., home visits and text reminders) and indirect (e.g., training of community leaders) communication approaches were described. In addition to limited access to information about vaccines in Africa, barriers to uptake included access to inoculations and high cost.

Among refugee subgroups, the included studies’ findings revealed that refugees benefitted from ample services that were delivered at various stages, that were not solely related to vaccinations, and that created multiple positive touch points with health and immigration systems. As with communication strategies across Africa, these approaches built trust and vaccine confidence through culturally responsive healthcare delivery. Culturally responsive approaches met patients where they were and provided readily accessible translation and on-site vaccinations, alongside educational and outreach partnerships with private groups and community organizations and leaders. These themes highlight the complexity of vaccine hesitancy and underscore the need for targeted public health interventions with culturally responsive messaging among diverse population subgroups.

The papers included in this Special Issue report both what we know and clarify what we still have to learn. The challenge of increasing the reach of vaccination worldwide remains. Yet, vaccine hesitancy is still not fully understood. Multi-level approaches—with individuals, parents, clinical teams, healthcare institutions, engaged community stakeholders, and policy-makers—have been found to be effective in increasing vaccine uptake [22]. These approaches have not been systematically implemented, however.

For future research on the factors influencing vaccine hesitancy and uptake worldwide, the range of vaccines studied, particularly among adolescents and high-risk groups, could be expanded to include Human Papillomavirus (HPV) vaccinations. Almost all cervical cancers are caused by persistent infections with oncogenic, or high-risk, types of HPV. Due to the rise in HPV-associated oropharyngeal (oral cavity and pharynx) cancer, in particular, the incidence of this cancer is rising, with men more than twice as likely as women to be diagnosed [23,24,25,26,27]. The HPV vaccine is effective at preventing more than 90% of HPV infection-associated cancers, however [28,29,30,31,32]. Yet, vaccination rates remain significantly below the World Health Organization’s goal of 90% inoculation [33].

More and varied use of population databases could lay the foundation for rigorous studies of HPV hesitancy, particularly with the possible widespread implementation of the single-dose HPV vaccine as recommended by the World Health Organization. With the variations in HPV vaccine implementation approaches worldwide, both researchers and healthcare system leaders should look for opportunities to perform pragmatic randomized clinical trials of interventional approaches to increase the evidence base. Implementation science models and approaches can further help to define the contexts within which effective approaches and their components could best be spread and sustained. Further, the continued development and validation of relevant measures of vaccine hesitancy could increase the generalizability of findings. 

As limited funding for vaccine programs and barriers to access affect their reach, implementation science approaches can inform future public health interventions. Those findings will lead to approaches for vaccines to reach those who are most vulnerable to the diseases that they prevent.

## References

[1] World Health Organization WHO. Vaccines and Immunization. https://www.who.int/health-topics/vaccines-and-immunization#tab=tab_1.

[2] World Health Organization Ten Threats to Global Health in 2019. https://www.who.int/news-room/spotlight/ten-threats-to-global-health-in-2019.

[3] Al-Qerem W., Jarab A., Eberhardt J., Alasmari F., Hammad A., Hour S.A. (2024). Acceptance of Flu Vaccine among Parents of Diabetic Children in Jordan. Vaccines.

[4] Pérez-Rivas F.J., Esteban-Gonzalo L., García-García D. (2024). Attitude Towards Vaccination Among University Students at a Spanish University: Relationships with Sociodemographic and Academic Variables. Vaccines.

[5] Chauhadry I.A., Soofi S.B., Sajid M., Ali R., Khan A., Naqvi S.K., Hussain I., Umer M., Bhutta Z.A. (2024). Bridging the Vaccination Equity Gap: A Community—Driven Approach to Reduce Vaccine Inequities in Polio High-Risk Areas of Pakistan. Vaccines.

[6] Chan P.S.-f., Poon J., Han S.C., Ye D., Yu F.-Y., Fang Y., Wong M.C.S., Mo P.K.H., Wang Z. (2024). Changes in the Pneumococcal Vaccination Uptake and Its Determinants before, during, and after the COVID-19 Pandemic among Community-Living Older Adults in Hong Kong, China: Repeated Random Telephone Surveys. Vaccines.

[7] Skyles T.J., Stevens H.P., Davis S.C., Obray A.M., Miner D.S., East M.J., Davis T., Hoelzer H., Piccolo S.R., Jensen J.L. (2024). Comparison of Predictive Factors of Flu Vaccine Uptake Pre- and Post-COVID-19 Using the NIS-Teen Survey. Vaccines.

[8] Aghajafari F., Wall L., Weightman A., Ness A., Lake D., Anupindi K., Moorthi G., Kuk B., Santana M., Coakley A. (2024). COVID-19 Vaccinations, Trust, and Vaccination Decisions within the Refugee Community of Calgary, Canada. Vaccines.

[9] Lee K.W., Yap S.F., Ong H.T., Liew S.L., Oo M., Swe K.M.M. (2024). COVID-19 Vaccine Booster Hesitancy among the Elderly in Malaysian Residential Care Homes: A Cross-Sectional Study in Klang Valley. Vaccines.

[10] Yue Z., McCormick N.P., Ezeala O.M., Durham S.H., Westrick S.C. (2024). EMSIG: Uncovering Factors Influencing COVID-19 Vaccination Across Different Subgroups Characterized by Embedding-Based Spatial Information Gain. Vaccines.

[11] Al-Qerem W., Jarab A., AlBawab A.Q., Hammad A., Alazab B., Abu Husein D., Eberhardt J., Alasmari F. (2023). Examining Influenza Vaccination Patterns and Barriers: Insights into Knowledge, Attitudes, and Practices among Diabetic Adults (A Cross- Sectional Survey). Vaccines.

[12] Peng Y., Wang Y., Huang W., Lin J., Zeng Q., Chen Y., Qiao F. (2024). Exploring Perceptions and Barriers: A Health Belief Model-Based Analysis of Seasonal Influenza Vaccination among High-Risk Healthcare Workers in China. Vaccines.

[13] Meites E., Szilagyi P.G., Chesson H.W., Unger E.R., Romero J.R., Markowitz L.E. (2019). Human Papillomavirus Vaccination for Adults: Updated Recommendations of the Advisory Committee on Immunization Practices. MMWR Morb. Mortal. Wkly. Rep..

[14] Batarfi S.A., Sutan R., Ismail H., Bin-Ghouth A.S. (2024). Immunization of Children under 2 Years Old in the Coastal Hadhramaut Governorate, Yemen, during Public Health Emergencies: A Trend Analysis of 2013–2020. Vaccines.

[15] Lamprinos D., Vroulou M., Chatzopoulos M., Georgakopoulos P., Deligiorgi P., Oikonomou E., Siasos G., Botonis P.G., Papavassiliou K.A., Papagiannis D. (2024). Influenza Vaccination Practices and Perceptions Among Young Athletes: A Cross-Sectional Study in Greece. Vaccines.

[16] Cao H., Chen S., Liu Y., Zhang K., Fang Y., Chen H., Hu T., Zhong R., Zhou X., Wang Z. (2024). Parental Hesitancy toward Seasonal Influenza Vaccination for Children under the Age of 18 Years and Its Determinants in the Post-Pandemic Era: A Cross-Sectional Survey among 1175 Parents in China. Vaccines.

[17] Zheng M., Du M., Yang G., Yao Y., Qian X., Zhi Y., Ma L., Tao R., Zhu Z., Zhou F. (2023). Mpox Vaccination Hesitancy and Its Associated Factors among Men Who Have Sex with Men in China: A National Observational Study. Vaccines.

[18] Qin C., Joslyn S., Han J.H., Savelli S., Agrawal N. (2024). Messaging to Reduce Booster Hesitancy among the Fully Vaccinated. Vaccines.

[19] Tan S.Y., Oka P., Tan N.C. (2023). Intention to Vaccinate against COVID-19 in Adolescents: A Systematic Review. Vaccines.

[20] Ekezie W., Igein B., Varughese J., Butt A., Ukoha-Kalu B.O., Ikhile I., Bosah G. (2024). Vaccination Communication Strategies and Uptake in Africa: A Systematic Review. Vaccines.

[21] Janz N.K., Becker M.H. (1984). The Health Belief Model: A decade later. Health Educ. Q..

[22] Rodriguez S.A., Mullen P.D., Lopez D.M., Savas L.S., Fernández M.E. (2019). Factors associated with adolescent HPV vaccination in the U.S.: A systematic review of reviews and multilevel framework to inform intervention development. Prev. Med..

[23] Parkin D.M., Bray F., Ferlay J., Pisani P. (2001). Estimating the world cancer burden: Globocan 2000. Int. J. Cancer.

[24] Muñoz N., Bosch F.X., de Sanjosé S., Herrero R., Castellsagué X., Shah K.V., Snijders P.J.F., Meijer C.J., International Agency for Research on Cancer Multicenter Cervical Cancer Study Group (2003). Epidemiologic classification of human papillomavirus types associated with cervical cancer. N. Engl. J. Med..

[25] Mendenhall W.M., Werning J.W., Pfister D.G., deVita V.T., Lawrence T.S., Rosenberg S.A. (2011). Treatment of head and neck cancer. Cancer: Principles and Practice of Oncology.

[26] Howlader N., Noone A.M., Krapcho M., Miller D., Brest A., Yu M., Ruhl J., Tatalovich Z., Mariotto A., Lewis D.R. (1975–2017). SEER Cancer Statistics Review.

[27] American Cancer Society (2024). Cancer Facts & Figures. https://www.cancer.org/research/cancer-facts-statistics/all-cancer-facts-figures/2024-cancer-facts-figures.html.

[28] Walker T.Y., Elam-Evans L.D., Singleton J.A., Yankey D., Markowitz L.E., Fredua B., Williams C.L., Meyer S.A., Stokley S. (2017). National, Regional, State, and Selected Local Area Vaccination Coverage Among Adolescents Aged 13–17 Years—United States, 2016. MMWR Morb. Mortal. Wkly. Rep..

[29] MMWR Morb Mortal Wkly Rep. https://www.cdc.gov/mmwr/volumes/67/wr/mm6741a8.htm.

[30] CDC Vaccination in Rural Communities as a Public Health Issue. https://www.cdc.gov/rural-health/php/public-health-strategy/public-health-strategies-for-vaccination-in-rural-communities.html.

[31] CDC TeenVaxView. https://www.cdc.gov/teenvaxview/index.html.

[32] U.S. Department of Health and Human Services Centers for Disease Control Prevention and National Cancer Institute U.S. Cancer Statistics: Data Visualizations. https://gis.cdc.gov/cancer/USCS/#/AtAGlance/value,1,2,1,1,1,1.

[33] PAHO/WHO Human Papillomavirus (HPV) Vaccine. https://www.paho.org/en/human-papillomavirus-hpv-vaccine.

